# 17β‐Oestradiol facilitates M2 macrophage skewing and ameliorates arrhythmias in ovariectomized female infarcted rats

**DOI:** 10.1111/jcmm.17344

**Published:** 2022-05-05

**Authors:** Cheng‐Che Lee, Syue‐yi Chen, Tsung‐Ming Lee

**Affiliations:** ^1^ Kang‐Ming Senior High School Tainan Taiwan; ^2^ Cardiovascular Institute An Nan Hospital China Medical University Tainan Taiwan; ^3^ 38019 Department of Medicine China Medical University Taichung Taiwan

**Keywords:** 17β‐oestradiol, macrophages, myocardial infarction, superoxide, sympathetic nerve

## Abstract

Epidemiological studies have suggested a lower incidence of arrhythmia‐induced sudden cardiac death in women than in men. 17β‐oestradiol (E2) has been reported to have a post‐myocardial infarction antiarrhythmic effect, although the mechanisms have yet to be elucidated. We investigated whether E2‐mediated antioxidation regulates macrophage polarization and affects cardiac sympathetic reinnervation in rats after MI. Ovariectomized Wistar rats were randomly assigned to placebo pellets, E2 treatment, or E2 treatment +3‐morpholinosydnonimine (a peroxynitrite generator) and followed for 4 weeks. The infarct sizes were similar among the infarcted groups. At Day 3 after infarction, post‐infarction was associated with increased superoxide levels, which were inhibited by administering E2. E2 significantly increased myocardial IL‐10 levels and the percentage of regulatory M2 macrophages compared with the ovariectomized infarcted alone group as assessed by immunohistochemical staining, Western blot and RT‐PCR. Nerve growth factor colocalized with both M1 and M2 macrophages at the magnitude significantly higher in M1 compared with M2. At Day 28 after infarction, E2 was associated with attenuated myocardial norepinephrine levels and sympathetic hyperinnervation. These effects of E2 were functionally translated in inhibiting fatal arrhythmias. The beneficial effect of E2 on macrophage polarization and sympathetic hyperinnervation was abolished by 3‐morpholinosydnonimine. Our results indicated that E2 polarized macrophages into the M2 phenotype by inhibiting the superoxide pathway, leading to attenuated nerve growth factor‐induced sympathetic hyperinnervation after myocardial infarction.

## 1 INTRODUCTION

Epidemiological studies have suggested that premenopausal women have a lower incidence of cardiovascular diseases and the associated mortality and morbidity compared to age‐matched men, but that this effect is not found post menopause.^1^ Women appear to have a lower incidence of arrhythmia‐induced sudden cardiac death than men.^2^ Women appear to have a better long‐term survival compared with men after a myocardial infarction (MI).^3^ The mechanisms by which gender affects arrhythmias are not well understood. The antioxidative effects of hormones may play a significant role in protecting cardiomyocytes against oxidative stress.^4^ Oestrogen has been shown to upregulate the level and activity of antioxidant enzymes in the myocardium.^5^ Furthermore, oestradiol (E2) has been shown to decrease activation of reactive oxygen species (ROS) production system, such as p47phox which reduced superoxide production.^6^

Following MI, inflammation is a critical pathological process. Macrophages are important inflammatory cells during ventricular remodelling after MI. Macrophages are special immune cells with high plasticity and different functional profiles. The pro‐inflammatory M1 macrophages secrete cytokines, while the anti‐inflammatory M2 macrophages promote tissue repair.^7^ M2 macrophages can be categorized into M2a, M2b, M2c and M2d subtypes.^8^ Prolonged exposure to M1 macrophages can increase infarct size and hinder scar formation and the resolution of inflammation.^9^ The dampening of the inflammatory response is associated with the M2c phenotype, characterized by the production of IL‐10.^8^ The selective suppression of M2‐like macrophages by deleting the *Trib1* gene after MI resulted in a reduced survival rate compared with littermates.^10^ Post‐MI cardiac remodelling was regulated by the M1/M2 balance.^11^, ^12^

There exists an association between macrophage polarization and sympathetic sprouting. Macrophage may promote axonal sprouting and hyperinnervation by secreting numerous biologically active molecules. Macrophages are an important source of nerve growth factor (NGF) following injury.^13^ A reduction in the number of macrophages has been shown to significantly decrease the level of NGF and impede sympathetic reinnervation post‐MI.^14^ The trophic factor NGF has been reported to play a crucial role in survival, differentiation, and synaptic activity of peripheral sensory nervous and sympathetic systems.^15^ The expression of NGF within innervated tissues has been shown to be approximately correlated with innervation density.^16^ In addition, the deletion of a single copy of the *NGF* gene has been reported to result in a 50% decrease in the number of sympathetic neurons,^17^ while an overexpression of NGF in the heart has been shown to lead to cardiac hyperplasia and hyperinnervation in neurons of the stellate ganglia.^18^ Taken together, these findings highlight the critical role that the macrophage‐NGF pathway plays in regulating sympathetic innervation.

A post‐MI elevation in sympathetic nerve density has been demonstrated to cause sudden cardiac death and fatal arrhythmias in humans.^19^ Moreover, regional increases in sympathetic nerves (neural remodelling) have frequently been observed during the chronic stage of MI.^20^ Furthermore, an increase in sympathetic nerve activity has been shown to play a critical role in the development of sudden cardiac death and ventricular arrhythmias.^19^ Attenuated sympathetic reinnervation after MI by ablation of cardiac sympathetic neurons has been shown to improve ventricular electrical remodelling.^21^ Very recently, we have shown that antioxidants can polarize macrophages to M2 through activation of the STAT3.^22^ Studies on the specific role that macrophages play in hyperinnervation are scarce, even though macrophages have been demonstrated to modulate injured peripheral nerve regeneration. Considering that motifs that resemble a classical oestrogen response have been identified in the 5′‐flanking and promoter regions of the NGF receptor gene,^23^ we hypothesized that oestrogen may regulate the biological effects and expression of NGF. Whether oestrogen may remodel sympathetic nerve by attenuated ROS‐mediated axis of macrophage‐NGF remained unknown. In this study, we assessed (1) whether the rescue administration of E2 could attenuate sympathetic innervation through the regulation of macrophage phenotypes, and (2) whether superoxide was involved in E2 protection using pharmacological interventions in a rat MI model.

## 2 MATERIAL AND METHODS

### 2.1 Ethics

All experiments were conducted according to the NIH guidelines for the care and use of animals. The Institutional Review Committee of our institution approved the procedures for animal care, surgery and euthanasia.

### 2.2 Animals

**FIGURE 1:**
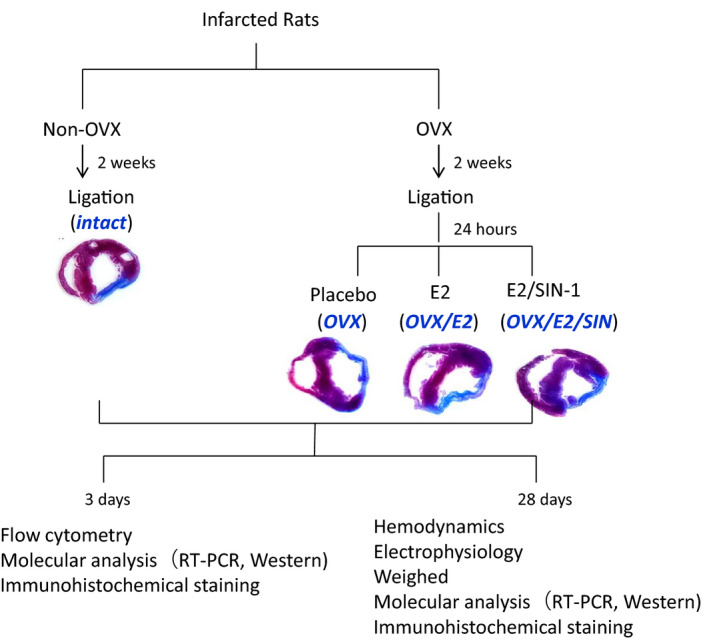
Grouping and flow chart of the study

Female Wistar rats that were approximately 8 weeks of age and weighed 120–150 g were anaesthetized with Zoletil (20 mg/kg body weight, Virbac AH, Inc., France) and xylazine (9 mg/kg), and subsequently underwent ovariectomies (OVX) via bilateral dorsal incisions. Two weeks later, slow‐release 17β‐E2 pellets were implanted subcutaneously in the dorsal neck area (0.25 mg 60‐day release pellets, Innovative Research of America, Sarasota, Florida) 24 h after coronary ligation. These pellets ensured physiologic E2 plasma concentrations and prevented fluctuations in E2 level due to the oestrus cycle. Rats that did not undergo OVX and the other rats that did undergo OVX received placebo pellets. Oestrogen status was confirmed according to the weight of the uterus and plasma E2 level. To confirm the importance of the ROS signalling in E2‐induced macrophage skewing, we employed 3‐morpholinosydnonimine (2.5 mg/kg per day once daily, SIN‐1, a peroxynitrite generator; Sigma‐Aldrich, St. Louis, MO, USA). Thus, a total of five experimental groups were studied: sham group and infarction groups (non‐OVX intact, OVX, OVX/E2, and OVX/E2/SIN‐1, Figure 1). The heart was excised at Day 3 or 28 after MI as early and late stages of MI.

### 2.3 Haemodynamics and Infarct size measurements

At the end of the study, the size of the infarcts and hemodynamic parameters were measured (online Supplementary material). Only rats with clinically important MIs (>30%) were selected for analysis.^24^

### 2.4 Ex vivo electrophysiological studies

We then performed programmed electrical stimulation to evaluate the possible arrhythmogenic risk of sympathetic innervation. We used the Langendorff heart perfusion technique in order to avoid any potential confounding effects of post‐MI hormonal activation on pacing‐induced ventricular arrhythmias as previously reported.^25^ Modified Tyrode's solution was used to perfuse the hearts at a constant flow rate of 4 ml/min and temperature of 37°C. Ventricular and atrial epicardial electrocardiograms were continuously recorded. After the hearts had been perfused, they were observed for 10 min to allow the contraction and rhythm to stabilize. A Bloom stimulator (Fischer Imaging Corporation, Denver, CO, USA) was used to generate pacing pulses, with a 120‐ms cycle length (S_1_) for eight beats, followed by one to three extrastimuli (S_2_, S_3_ and S_4_) at shorter coupling intervals to induce ventricular arrhythmias. The induction of ventricular tachyarrhythmia was defined as being the endpoint of ventricular pacing. Nonsustained ventricular tachyarrhythmias including ventricular fibrillation and tachycardia were considered to be those that lasted for ≤15 beats, and sustained ventricular tachyarrhythmias were considered to be those that lasted for >15 beats. An arrhythmia scoring system was modified as previously described.^26^ 0, noninducible; 1, nonsustained tachyarrhythmias induced with three extrastimuli; 2, sustained tachyarrhythmias induced with three extrastimuli; 3, nonsustained tachyarrhythmias induced with two extrastimuli; 4, sustained tachyarrhythmias induced with two extrastimuli; 5, nonsustained tachyarrhythmias induced with one extrastimulus; 6, sustained tachyarrhythmias induced with one extrastimulus; 7, tachyarrhythmias induced during the eight paced beats; and 8, the heart stopped before the pacing. When multiple forms of arrhythmias occurred in one heart, the highest score was used. The experimental protocols were typically completed within 10 min. Detailed information is provided in the online Supplementary material.

### 2.5 Flow cytometry

Flow cytometry was used to quantitatively analyse the number of left ventricular (LV) macrophages and evaluate the phenotype from tissue digests on post‐MI Day 3. The LV was dissected from the border zone (<2 mm away from the infarct) after any residual blood had been rinsed off. The samples were then digested in DNase, collagenase IV and hyaluronidase at 37°C for 30 min, filtered through 70‐μm nylon mesh and centrifuged for 5 min at 600 *g*. The cell suspensions were then washed and blocked using anti‐CD32/CD16 antibodies before staining. CD45^+^ and CD11b^+^F4/80^+^ cells were identified as M1 macrophages, and CD45^+^ and CD206^+^ F4/80^+^ cells were identified as M2 macrophages. A flow cytometer (Accuri C6; BD Biosciences, San Jose, CA, USA) was used to assess fluorescence labelling.

### 2.6 Western blot analysis of *iNOS*, *IL*‐*10* and *NGF*

iNOS and IL‐10 were evaluated in samples obtained from the border zone on Day 3, and NGF was evaluated in samples obtained from the remote zone (>2 mm away from the infarct) on Day 28. All of the experiments were performed in triplicate, and the results were expressed as mean values. For a detailed information, please refer to the Supplementary material online.

### 2.7 Immunohistochemical studies of *CD68*, *iNOS*, *IL‐10*, *CD11b*, *CD206*, *NGF*, *DHE*, tyrosine hydroxylase, growth associated factor 43 and neurofilament

To assess the macrophage polarization after MI, immunohistochemical staining was performed in the border zone at Day 3 for CD68 (a marker for all macrophages, Abcam, Cambridge, MA), iNOS (a marker for M1, Cell Signaling Technology, Danvers, MA, USA) and IL‐10 (a marker for M2c, R& D systems, Abingdon, UK). To assess the contribution of different macrophage phenotypes to the NGF content, co‐staining for macrophages (CD11b as a marker for M1 and CD206 as a marker for M2) and NGF was performed from samples in the border zone at Day 3.

Myocardial intracellular superoxide production was evaluated using *in situ* dihydroethidium (DHE; Invitrogen Molecular Probes, Eugene, OR, USA) fluorescence, after OCT‐embedded tissues had been incubated with DHE from the border zone on post‐MI Day 3. To minimize interference caused by nonspecific DHE oxidation products, red fluorescence was detected with excitation from 480–405 nm.^27^

To quantify sympathetic nerve fibres and spatial distribution, immunohistochemical staining was performed for tyrosine hydroxylase, growth associated factor 43 (a neuronal regeneration and outgrowth marker peptide) and neurofilaments (a sympathetic nerve marker) on post‐MI Day 28 remote region LV muscle tissue. The tissue was incubated with anti‐tyrosine hydroxylase antibodies (1:200; Chemicon, CA, USA), anti‐growth associated factor 43 antibodies (1:400; Chemicon, CA, USA) and anti‐neurofilament antibodies (1:200; Chemicon, CA, USA). The average of 10 random scans per section was used for analysis. Quantification was calculated as positively stained area/total area (%) at 400× magnification.

### 2.8 Real‐time PCR of *IL‐6*, *IL‐1β*, *iNOS*, *CD206*, *IL‐10* and *NGF*

Real‐time polymerase chain reaction (PCR) was performed using samples obtained at Day 3 for *IL‐6*, *IL‐1β*, *iNOS*, *CD206* and *IL‐10* from the border zone, and at Day 28 for *NGF* from the remote zone using a TaqMan system (Prism 7700 Sequence Detection System, PE Biosystems) as previously described.^25^ The gene expression patterns for M1 (*IL*‐*6*, *IL*‐*1β*, *iNOS*) and M2 (*CD206*, *IL*‐*10*) macrophages were assessed. *Cyclophilin A* mRNA was used as the internal standard as it is present at a reasonably constant level in most tissues. The reaction conditions were implemented for 40 cycles of the amplification step using a computer that was connected to the detector. The PCR primers sequences are shown in the online data supplement.

### 2.9 Laboratory measurement

Blood samples were separated by centrifugation at 3000*g* for 10 min at 4°C. E2 levels were measured using a commercial kit (Sigma, St. Louis, MO).

Lucigenin‐enhanced chemiluminescence (5 μM, bis‐N‐methylacridinium nitrate, Sigma, St. Louis, MO) was used to measure myocardial superoxide production from the border zone as previously described.^28^ Specific chemiluminescence signals were obtained by subtracting background activity and expressed as counts per minute per milligram weight (cpm/mg).

Myocardial IL‐10 activity for M2c was assayed from the border zones, and the myocardial membrane‐bound IL‐10 fraction was evaluated using a commercially available ELISA kits (R&D Systems). Norepinephrine levels of the supernatant proteins from the remote zone were also evaluated using a commercially available ELISA kit (Noradrenalin ELISA, IBL Immuno‐Biological Laboratories Co., Hamburg, Germany).

### 2.10 Statistical analysis

All statistical analyses were performed using SPSS version 23 (SPSS, Chicago, Illinois), and results were presented as mean ± SD. Differences among the groups of rats were tested using one‐way ANOVA. Subsequent analysis for significant between‐groups differences was performed using a multiple comparison test (Scheffe's method). Electrophysiological results (programmed electrical stimulation‐induced arrhythmia scores) were compared using the Kruskal–Wallis test followed by the Mann–Whitney test. A *p* value < 0.05 was considered to indicate statistical significance.

## 3 RESULTS

### 3.1 Part 1: acute stage (Day 3)

#### 3.1.1 E2 attenuated oxidative stress

**FIGURE 2:**
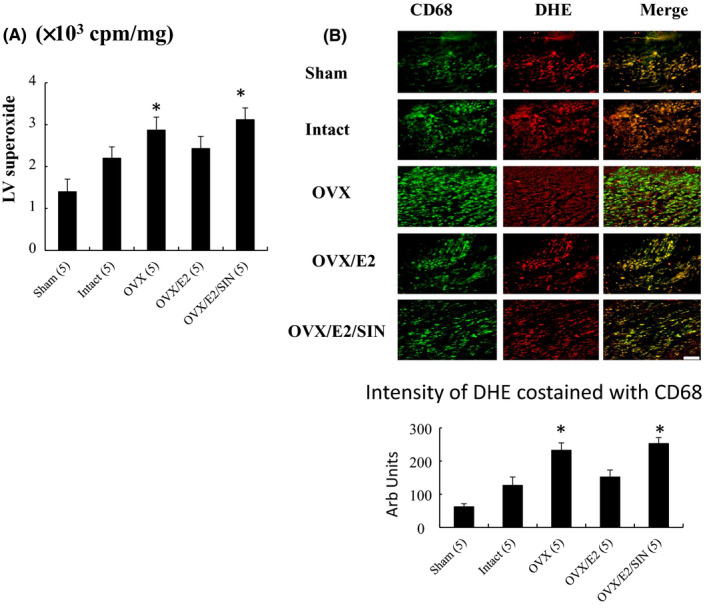
*In vivo* myocardial superoxide levels in the border zone at Day 3 after MI. Myocardial (A) superoxide levels were assessed using chemiluminescence and (B) DHE costained with a CD68 macrophage marker and quantitative analysis. Myocardial DHE (red fluorescence) staining showed more intense signals after MI. The number of animals in each group is indicated in parentheses. Bar = 50 μm. *p < 0.05 compared with the sham and infarcted groups treated with intact and OVX/E2

Lucigenin‐enhanced chemiluminescence revealed a marked post‐MI increase in myocardial superoxide production in the border zone compared to the sham group (*p* = 0.012, Figure 2A). In addition, there was a significantly lower level of superoxide in the intact‐ and E2‐treated infarcted groups compared to the OVX group. However, this effect of attenuating superoxide was not seen after the administration of SIN‐1. These results were further confirmed by DHE staining (Figure 2B).

#### 3.1.2 E2 regulated macrophages towards a M2 phenotype through superoxide signalling

**FIGURE 3:**
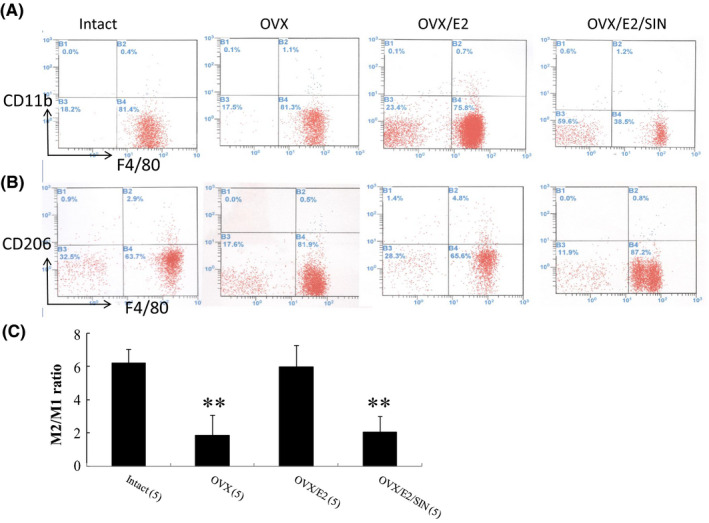
Flow cytometry analyses of macrophages in the border zone at Day 3 after MI. Macrophage accumulation was seen in the border zone. E2 induced a phenotypic switch in macrophage polarization and significantly reduced the M1 percentage and increased the M2 percentage, resulting in an increase in the M2/M1 ratio. (A, B) Representative flow cytometry plots showing macrophage distribution by CD11b (A) and CD206 (B). (C) The ratio of M2/M1 in the myocardium. The number of animals in each group is indicated in parentheses. *p < 0.05, **p < 0.01 versus infarcted groups treated with intact and OVX/E2

Flow cytometry analysis of infarcted heart cells revealed two macrophage subsets: M1 macrophages characterized by staining for both CD11b and F4/80, and M2 macrophages characterized by staining for both CD206 and F4/80. OVX increased the percentage of M1 macrophages by 3.34‐fold compared to the intact group (Figure 3, *p* = 0.0052). The administration of E2 significantly reversed this skewing effect and increased the M2/M1 ratio compared to the OVX group (*p* = 0.0032).

**FIGURE 4:**
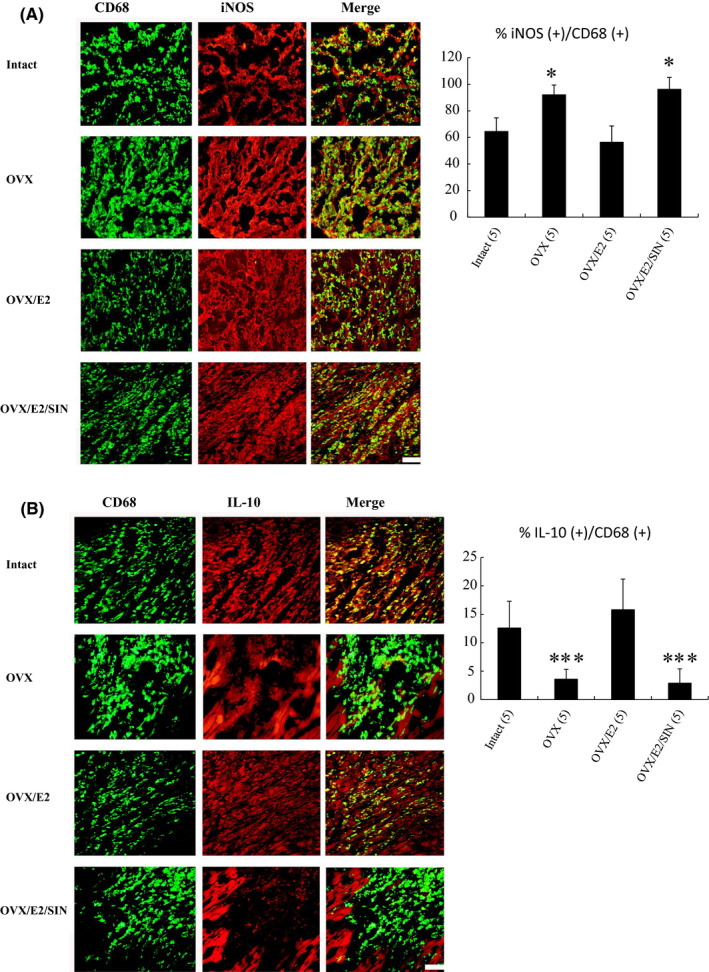
Immunohistochemical staining of M1 and M2 macrophage phenotype in the border zone at Day 3 after MI. A, iNOS‐expressing CD68^+^ M1 macrophages were recruited to the infarcted myocardium in the intact group, but were significantly reduced after E2 administration. The M1 ratio was significantly higher in the OVX group than in the OVX/E2 group. B, IL‐10‐expressing CD68^+^ M2 macrophages were predominant in E2‐administered infarcted myocardium. The E2‐treated group had a significantly higher M2 ratio compared to the OVX group. The iNOS‐expressing CD68^+^ or IL‐10‐expressing CD68^+^ macrophages were calculated and expressed as bar graphs. The number of animals in each group is indicated in parentheses. Bar = 50 μm. *p < 0.05, ***p < 0.001 versus infarcted groups treated with intact and OVX/E2

To confirm the flow cytometry findings, we assessed the effect of E2 on macrophage differentiation by examining type‐specific surface markers. Markers for M1 (CD68^+^, iNOS^+^) and M2c (CD68^+^, IL‐10^+^) were examined to evaluate the subtypes of infiltrated macrophages in the infarcted myocardium (Figure 4). Immunohistochemical staining demonstrated infiltration of CD68^+^ macrophages in the infarcted groups on post‐MI Day 3. In addition, iNOS‐expressing CD68^+^ macrophages were significantly higher in the OVX group than in OVX/E2 group (92.1 ± 7.3% in the OVX group versus 56.3 ± 12.3% in the OVX/E2 group, *p* = 0.022, Figure 4A), and there were more IL‐10‐expressing CD68^+^ macrophages in the OVX/E2 group (3.6 ± 1.7% in the OVX group versus 15.8 ± 5.4% in the OVX/E2 group, *p* < 0.001, Figure 4B). These data suggested that the E2‐treated rats had a better ability to increase M2 macrophage differentiation while concomitantly reducing the number of M1 macrophages.

**FIGURE 5:**
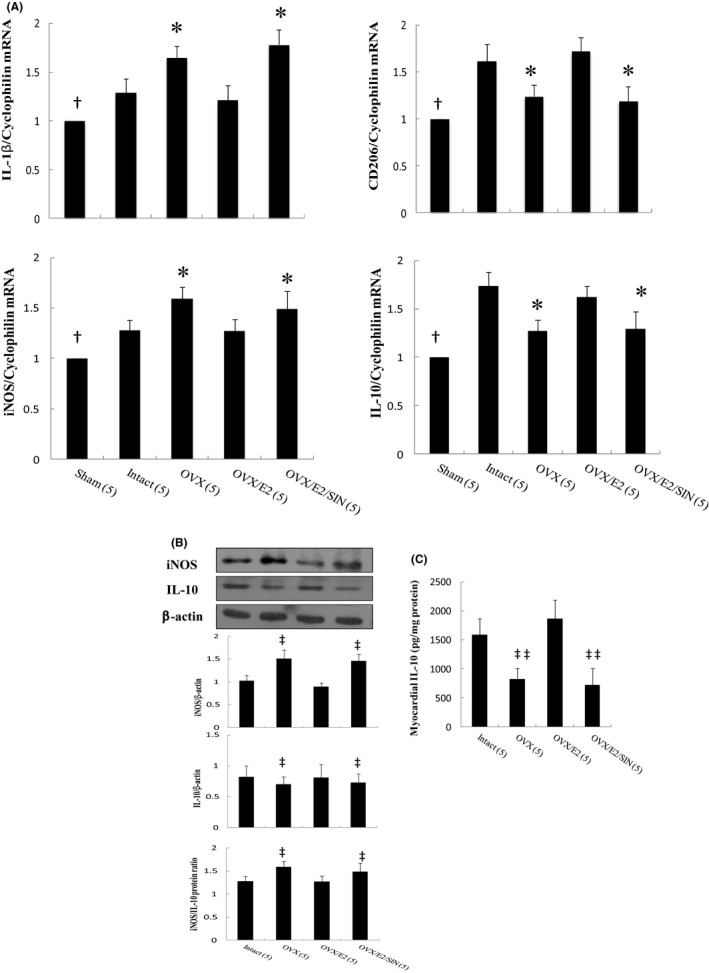
Expression of gene markers and function for M1 and M2 macrophages at Day 3 after MI. (A) mRNA, (B) iNOS and IL‐10 protein levels and (C) myocardial IL‐10 activity. There was an obvious shift towards the M2 macrophage phenotype in the infarcted groups treated with E2 as shown by changes in the expressions of reduced M1‐related genes (*IL*‐*1β*, *iNOS*) and iNOS protein levels and increased M2‐related genes (*CD206*, *IL*‐*10*) and IL‐10 protein levels. The number of animals in each group is indicated in parentheses. *p < 0.05, compared with the sham and infarcted groups treated with intact and OVX/E2; †*p* < 0.05, compared with the infarcted group treated with intact; ‡*p* < 0.05, ‡‡*p* < 0.01 compared with infarcted groups treated with intact and OVX/E2

To investigate whether the superoxide signalling pathway is required to polarize M2c macrophages, we assessed the effect of SIN‐1 on transforming macrophages into the M2c subtype using Western blot, RT‐PCR and ELISA. There was a significant increase in macrophage accumulation in the infarcted heart at Day 3 after MI (Figure 5A). E2‐treated infarcted rats showed a marked decrease in M1 mRNA (*IL*‐*1β*, *iNOS)* and a marked increase in M2 mRNA *(CD206*, *IL*‐*10)* compared with OVX infarcted rats (Figure 5A). However, this increase in the expression of the M2 gene in the E2 group was reduced after the administration of SIN‐1. We then examined the protein levels of iNOS and IL‐10 by Western blotting (Figure 5B), which showed a significant reduction in iNOS and marked increase in IL‐10 in the E2 group. However, these changes were reversed after the administration of SIN‐1. These results showed that E2 treatment had an inhibitory effect on M1 markers and a stimulatory effect on M2 markers. Myocardial IL‐10 activity was significantly higher (*p* = 0.0042) in the E2‐treated group than in the OVX group (Figure 5C). However, the activity of IL‐10 was significantly decreased after adding SIN‐1.

**FIGURE 6:**
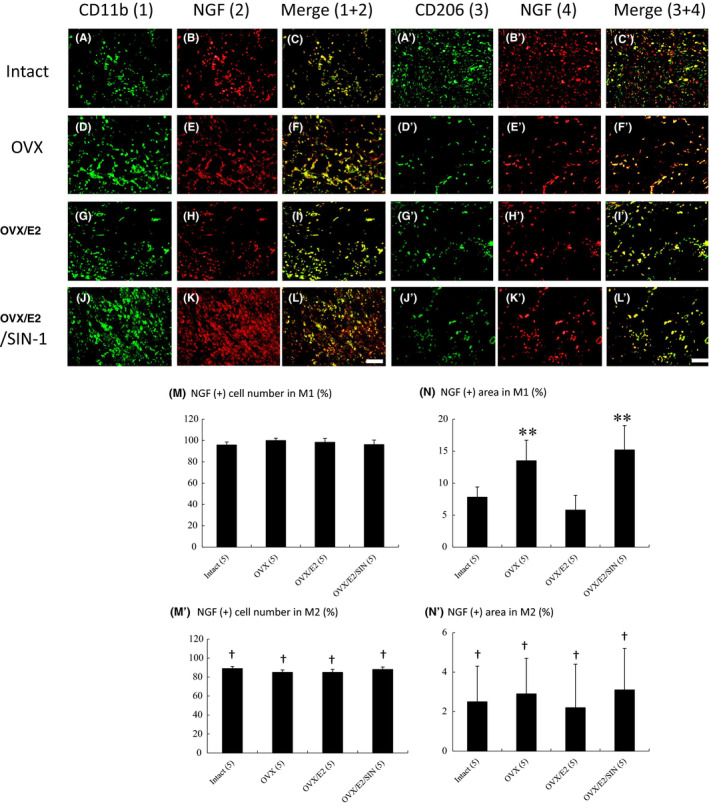
Effect of macrophage phenotypes on NGF expression at 3 days after MI. Immunostaining for CD11b (A, D, G, J) and CD206 (A′, D′, G′, J′) as a M1 and M2 macrophage markers, respectively, and costained for NGF (B, E, H, K, B′, E′, H′, K′), merged images (C, F, I, L, C′, F′, I′, L′). E2 administration attenuated the post‐MI M1 macrophages which had an increased content of NGF levels compared with M2 macrophages. Scale bar 50 μm. Quantitative analysis of macrophages expressing NGF as determined by the cell numbers and area of M1 (M, N) and M2 (M′, N′) coexpressing NGF. The number of animals in each group is indicated in parentheses. **p < 0.01 versus infarcted groups treated with intact and OVX/E2; †*p* < 0.05, compared with respective group in M1

To evaluate the effect of macrophage skewing on NGF expression, co‐immunostaining for NGF protein and macrophage markers was performed (Figure 6). Following MI, almost all CD11b^+^ macrophages co‐expressed NGF (Figure 6A‐N). However, CD206^+^ macrophages showed significantly lower co‐localization of NGF in cell number compared with CD11b^+^ macrophages (Figure 6M and M′). Furthermore, CD206^+^ macrophages showed significantly lower co‐localization of NGF in stained areas compared with CD11b^+^ macrophages (Figure 6N and N′). Thus, M2 macrophages have significantly lower NGF contents in terms of NGF‐stained cell number and areas compared with M1. After E2 supplement in OVX infarcted rats, CD11b^+^ macrophages express lower stained areas of NGF compared with OVX (Figure 6N). However, E2 did not affect NGF areas of M2 macrophages (Figure 6N′). Thus, E2 can decrease the NGF contents by 2 ways: macrophages skewing towards M2 and residual M1 with less NGF amount.

### 3.2 Part 2: chronic stage (Day 28)

#### 3.2.1 Body weight, E2 levels, uterus weight, infarct size, and haemodynamics

The haemodynamics and morphometry in all infarcted rats regardless of infarct size were shown in Table S1. Table 1 showed the data of the rats with infarct size >30%. The body weights of the infarcted rats receiving OVX were significantly higher than the other groups, even though there were no differences in body weight among any of the groups at baseline (Table 1). The finding that body weight was negatively associated with E2 status is consistent with a previous study.^29^

**TABLE 1:** Morphometry, haemodynamics, E2, and NE concentrations at the end of study

Parameters	Sham	Infarction treated with			
	Intact	Intact	OVX	OVX/E2	OVX/E2/SIN
No. of rats	10	10	12	11	10
Body weight, g	231 ± 20	228 ± 15	282 ± 25*,†	233 ± 18	229 ± 17
HR, bpm	402 ± 20	397 ± 21	433 ± 22*,†	405 ± 22	407 ± 25
LVESP, mmHg	105 ± 6	98 ± 8	107 ± 12	99 ± 12	96 ± 13
LVEDP, mmHg	5 ± 4	18 ± 4***	18 ± 5***	16 ± 5***	19 ± 6***
LVW/BW, mg/g	3.28 ± 0.27	3.48 ± 0.63	3.65 ± 0.49	3.39 ± 0.38	3.42 ± 0.37
RVW/BW, mg/g	0.42 ± 0.16	0.65 ± 0.17*	0.78 ± 0.18*	0.72 ± 0.21*	0.79 ± 0.15*
+d*p*/d*t*, mm Hg/s	7425 ± 227	2855 ± 224**	3727 ± 258**,†	3084 ± 265**	2778 ± 248**
−d*p*/d*t*, mm Hg/s	4762 ± 282	2542 ± 264*	2952 ± 242*,†	2549 ± 219*	2372 ± 253*
Infarct size, %	…	39.4 ± 3.6	40.5 ± 4.7	39.8 ± 4.3	40.2 ± 4.9
Uterus weight (mg/g body weight)	65 ± 12	69 ± 18	25 ± 12***,†††	84 ± 21	88 ± 17
E2 concentration, pg/ml	56.1 ± 12.3	68.3 ± 20.1	4.3 ± 1.5***,†††	92.5 ± 21.4	86.4 ± 18.3
NE, µg/g protein	1.10 ± 0.39	2.62 ± 0.28**	3.46 ± 0.38***,‡	2.25 ± 0.39**	3.85 ± 0.43***,‡

Note

Values are mean ± SD. BW, body weight; E2, oestradiol; HR, heart rate; LVEDP, left ventricular end‐diastolic pressure; LVESP, left ventricular end‐systolic pressure; LVW, left ventricular weight; NE, norepinephrine; OVX, ovariectomy; and RVW, right ventricular weight.

^*^p < 0.05, **p < 0.01, ***p < 0.001 compared with sham; ^†^ *p* < 0.05, †††*p* < 0.001 compared with infarcted groups treated with intact, OVX/E2, and OVX/E2/SIN; ^‡^ *p* < 0.05 compared with infarcted groups treated with intact and OVX/E2.

The OVX group that received an E2 pellet had a similar average plasma E2 level to that at pro‐oestrus in the cycling rats (average 92.5 ± 21.4 pg/ml). In contrast, the OVX group that received a placebo had a low oestrogen level (4.3 ± 1.5 pg/ml), which is similar to that in postmenopausal women. In addition, the uterine horn and body weight were significantly lower in the OVX group (25 ± 12 mg/g body weight) than in the sham‐operated intact group (65 ± 12 mg/g body weight).

There were no significant differences in the mean size of the infarcts or mortality among the infarcted groups. In addition, cardiac gross morphology was little affected by E2 in the sham‐operated group (data not shown). Four weeks after infarction, the weight of the LV inclusive of the septum remained relatively unchanged.

In the sham group, LVEDP, LVESP, +d*P*/d*t* and −d*P*/d*t* were not affected by E2 status (data not shown). The heart rate of the infarcted rats that underwent OVX was significantly higher than the intact, OVX/E2 and OVX/E2/SIN‐1 groups (Table 1). There were similar increases in LVEDP in the infarcted groups compared to the sham group.

#### 3.2.2 Sympathetic innervation

The levels of LV norepinephrine in the remote zone were significantly upregulated by 2.4‐fold in the non‐OVX infarcted group than in the sham‐operated group (2.62 ± 0.28 vs. 1.10 ± 0.39 µg/g protein, *p* = 0.0022, Table 1). Compared to the rats that underwent OVX, the administration of E2 resulted in a significant decrease in LV norepinephrine levels, and this effect was reversed after the administration of SIN‐1.

**FIGURE 7:**
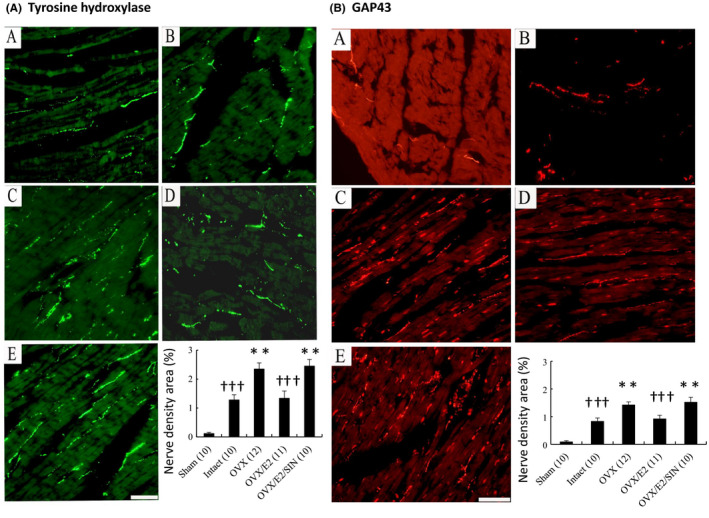
Sympathetic innervation at 28 day after MI. A, immunohistochemical staining for tyrosine hydroxylase from the remote regions (magnification 400×). Tyrosine hydroxylase‐positive nerve fibres were located between myofibrils and were oriented in the same longitudinal direction as the myofibrils. B, immunohistochemical staining for growth associated protein 43 from the remote regions (magnification 400×). The number of animals in each group is indicated in parentheses. Bar = 50 μm. A, sham; B, non‐OVX infarcted rat treated with placebo pellets (intact); C, OVX infarcted rat treated with placebo pellets (OVX); D, OVX infarcted rat treated with E2 (OVX/E2); E, OVX infarcted rat treated with E2 + SIN‐1 (OVX/E2/SIN). **p < 0.01 versus infarcted groups treated with intact and OVX/E2; †††*p* < 0.001 versus sham

The fraction of the area of the nerves that was positive for tyrosine hydroxylase was significantly larger in the intact infarcted group than in the sham group (Figure 7A). In addition, the infarcted E2‐treated group had a smaller nerve area fraction in the remote regions than the OVX group (1.34 ± 0.25% in the E2 group vs. 2.35 ± 0.21% in the OVX group, *p* = 0.0058). Similarly, there were significant attenuations in the fractions of nerve areas positive for growth associated factor 43 (Figure 7B) and neurofilament (data not shown) in the E2‐treated infarcted group compared to the OVX group. All of these attenuated expressions were reversed to levels similar to the OVX group after the administration of SIN‐1. These morphometric results were consistent with those for norepinephrine.

#### 3.2.3 Western blot and real‐time PCR of NGF

**FIGURE 8:**
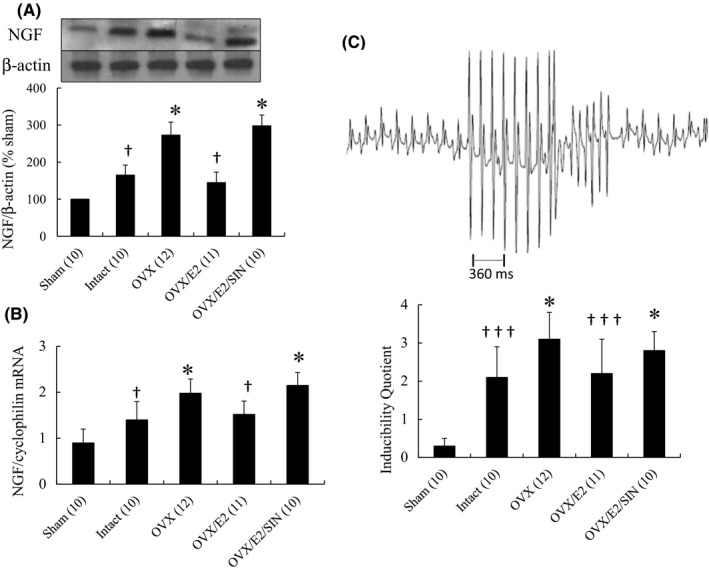
NGF protein and mRNA and arrhythmias at 28 day after MI. A, Western blot analysis showed the effect of E2 on immunorecognition of NGF in homogenates after MI from the remote zone. A significantly reduced amount of NGF was noted in the OVX group treated with E2 compared with OVX alone. Results are mean ± SD of 3 independent experiments. B, LV *NGF* mRNA levels. Each mRNA was corrected for an mRNA level of cyclophilin. C, Upper panel, a representative nonsustained ventricular arrhythmia induced by ventricular pacing in an infarcted rat treated with OVX. After eight basic stimuli at a cycle length of 120 ms, nonsustained ventricular tachyarrhythmia induced with one extrastimulus was observed (score: 5); Lower panel, inducibility quotient of ventricular arrhythmias by programmed electrical stimulation 4 weeks after MI. The number of animals in each group is indicated in parentheses. Each column and bar represents mean ± SD. *p < 0.05 versus infarcted groups treated with intact and OVX/E2; †*p* < 0.05, †††*p* < 0.001 versus sham

Western blot analysis showed significantly upregulated levels of NGF in the remote zone by 1.65‐fold in the intact infarcted group than in the sham‐operated group (*p* = 0.031, Figure 8A). In addition, NGF levels in the remote zone were significantly lower in E2‐treated group than in the OVX group. This attenuated expression of E2‐related NGF could be reversed to levels similar to the OVX group after the administration of SIN‐1.

PCR cDNA amplification revealed a 1.56‐fold upregulation of *NGF* mRNA levels in the remote zone in the non‐OVX infarcted group compared to the sham‐operated group (*p* = 0.029, Figure 8B). In addition, the *NGF* mRNA levels were significantly lower in the E2‐treated infarcted group than in the OVX‐treated group.

#### 3.2.4 Electrophysiological stimulation

We then performed ventricular pacing to further investigate the physiological effect of attenuated sympathetic reinnervation. The results showed a very low arrhythmia score in the sham‐operated group (0.3 ± 0.2, Figure 8C), whereas ventricular tachyarrhythmias consisting of ventricular tachycardia and ventricular fibrillation were inducible by programmed stimulation in the intact infarcted group. Moreover, the administration of E2 significantly reduced ventricular tachyarrhythmia inducibility compared to the OVX group. This beneficial effect of E2 on arrhythmic score was reversed by administering SIN‐1.

## 4 DISCUSSION

Our present results clearly show for the first time that despite the late administration of oestradiol 24 h after coronary ligation, chronic treatment with E2 at physiologic concentrations leads to attenuated sympathetic innervation by targeting M2 macrophages after MI. Our results extended previous studies, showing that oestrogen affects neurite outgrowth by modulating synthesis and release of neurotrophins.^30^ Macrophages are an important source of NGF. NGF was synthesized by infiltrating CD11b and CD206 immunopositive macrophages. Furthermore, CD11b^+^ macrophages had higher NGF contents. E2 polarized macrophages towards M2, which had less NGF contents. These results were consistent with the beneficial effects of E2, as seen structurally by M1/M2 polarization and cardiac nerve sprouting, molecularly by myocardial NGF mRNA and protein levels, biochemically by tissue superoxide and norepinephrine levels, pharmacologically by a ROS donation, and electrophysiologically by improvement in fatal ventricular tachyarrhythmias. Our results confirmed the finding that oestrogen shortens the duration of the pro‐inflammatory phase and favours the progression of macrophages towards the IL10‐dependent resolution phase.^31^ Moreover, our findings are consistent with previous studies, in that oestrogen reduced the expression of NGF protein in sympathetic vascular targets, and this may have led to a decrease in sympathetic innervation to the heart.^32^

**FIGURE 9:**
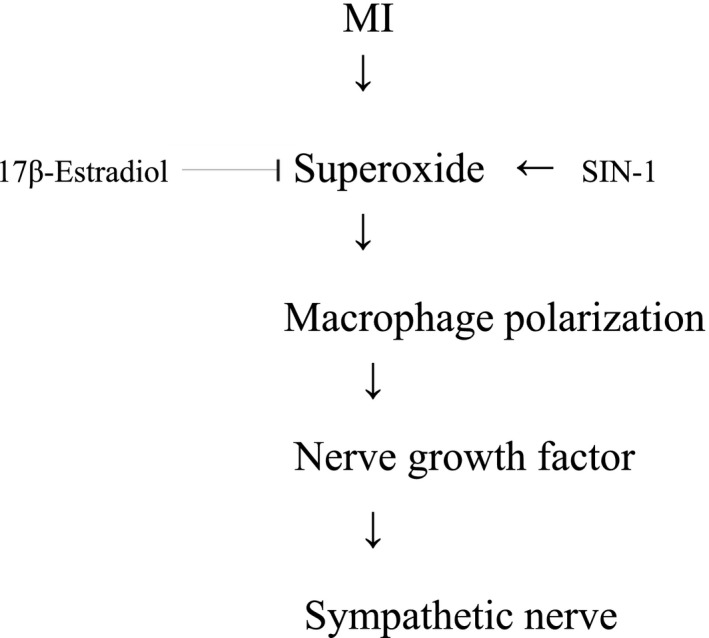
Reaction sequences leading to attenuated arrhythmias. The diagram summarizes the immunofluorescent, molecular and pharmacological evidence presented in this report. The inhibition of these signalling pathways by their respective inhibitors is indicated by the vertical lines

The beneficial effect of E2 on the anatomical and functional conditions of sympathetic reinnervation was supported by 3 lines of evidence as follows (Figure 9).

1. E2 administration attenuated superoxide levels after infarction. These results confirm our previous study of a significant increase in superoxide level after infarction,^33^ and that this could be inhibited after the administration of E2. However, the beneficial effects of E2 were not seen after the addition of SIN‐1, implying the important role of ROS in regulating E2‐mediated effects.
2. E2 administration amplified the post‐MI augmentation of cardiac M2 macrophages. E2 treatment amplified (4.4‐fold, Figure 4B) the post‐MI increase in M2 macrophages in the damaged myocardium compared with that seen in the OVX.
3. Macrophages induced NGF. NGF is associated with a mass accumulation of infiltrated macrophages. NGF is a key regulator of the cross‐talk between the macrophage and nervous systems. Previous studies have shown that NGF levels were significantly increased when M2 macrophages appeared in mice intervertebral discs following intervertebral disc injury.^34^ However, our study showed the NGF contents in M2 were significantly lower than those in M1. The discrepancy can be explained at least in part by the samples measured. Prior studies measured NGF levels from all the tissues of intervertebral discs, not M2 macrophages. Many cells produced NGF such as activated lymphocytes, eosinophils and mast cells,^35^ all of which were infiltrated into the injured discs.

### 4.1 Other mechanisms

Although our results suggest that the mechanisms by which E2 induces neuroprotection are related to the attenuation of NGF expression, oestrogen has been shown to have many effects on the heart, and other potential mechanisms such as electrical remodelling need to be investigated. E2 has been reported to potentially prevent fatal arrhythmias through the direct inhibition of electrophysiological alterations.^36^, ^37^, ^38^, ^39^ Both our group and other researchers have shown that E2 treatment can activate ATP‐sensitive potassium channels,^36^, ^37^ inhibit calcium channels,^38^ and downregulate the expression of Kv4.3,^39^ all of which play important roles in the induction of abnormal automaticity and reentrant arrhythmias. Stopping ionic remodelling may therefore be an upstream approach to treat arrhythmias.

### 4.2 Clinical implications

Our results suggest that modification of the myocardial environment by polarized macrophages mediated by E2 supplement is an exciting new approach for the treatment of post‐MI reinnervation. This study identifies a potential benefit of postmenopausal E2 replacement therapy: macrophage polarization. E2 plasma levels were low but still detectable, similar to the menopausal situation. To mimic the menopausal situation, the length of time between OVX and oestrogen administration, referred to as ‘washout period’, was set at 2 weeks. This skewing may be especially important in the presence of pathology where inflammation plays an etiological role, such as hypertension, diabetic nephropathy and atherosclerosis. The results of randomized primary prevention trials have demonstrated that giving hormone replacement therapy to postmenopausal women did not reduce sudden cardiac death and in certain circumstances caused harm.^40^, ^41^ In these trials, the women were given conjugated equine oestrogens with the addition of a progestogen for those with an intact uterus. While some of the adverse effects may be attributed to the progestogen component, the patients receiving conjugated equine oestrogens alone also failed to gain any cardiac benefit from this treatment.^40^ Although conjugated equine oestrogens are very effective in controlling menopausal symptoms such as hot flushes, they do not contain 17β‐oestradiol which is the main endogenous female sex hormone.^42^ A major difference between our study and previous clinical trials is that we used the chemical form of oestrogen, which may have had a major impact on its effects.

### 4.3 Study limitations

There are limitations to this study with regard to extending our results to other species and human physiology. First, steroid hormones can exert biphasic dose–response effects^43^ and the cardioprotective effects of oestrogens may be reversed at higher doses/plasma concentrations. The antioxidant effects of oestrogens may be prevalent at physiologic concentrations as shown in this study, whereas a higher concentration may increase the production of radicals and damage and lead to worsened cerebral ischaemia.^44^ Thus, oestrogens could be protective at physiological doses but harmful at pharmacological doses. Our results cannot be extended to the general use of oestrogens. Second, although other studies have reported that oestrogen can induce an increase in NGF, this increase has been reported in the uterus following an acute dose of oestrogen^30^ or in infantile/prepubertal rats subjected to chronic oestrogen replacement.^45^ The effects of oestrogen on NGF content are dependent on a number of different conditions, such as the target tissue investigated and the dose and duration of oestrogen replacement. Third, we do not know whether blocking oestrogen receptors using specific oestrogen receptor antagonists (e.g. ICI 182,780) will have a detrimental effect on cardiac innervation. Our findings do not provide information on the relative roles of the ERα or ERβ pathways. Fourth, our study does not provide a direct insight into distinct intracellular signalling cascades of M1 and M2. Previous studies have shown that differential effects of polarized macrophages on nerve regeneration: M1 macrophages have been shown to be neurotoxic and M2 macrophages have been shown to promote a regenerative growth response in adult sensory axons in mice with traumatic spinal cord injuries.^46^ Future studies are required to elucidate macrophage polarization in NGF contents and signalling pathways. Fifth, although NGF is well known for sympathetic hyperinnervation after MI, it is clear that NGF effects are more wide ranging. Recent studies reported both *in vitro* and *in vivo* evidence for beneficial actions of NGF on cardiomyocytes in normal and pathological hearts, including contractility effects.^47^ Although targeted intracardiac administration of NGF small interfering RNA reduced nerve sprouting and decreased sympathetic nerve density in a rat MI model, the effects of attenuated angiogenesis, augmented infarct size and exacerbated cardiac dysfunction on adverse ventricular remodelling were observed.^48^ Thus, the improvement of ventricular arrhythmias after administering E2 did not imply the beneficial effect on adverse ventricular remodelling and the relation between ventricular remodelling and arrhythmias was more complex than previously thought. Finally, one issue not fully resolved in this study is whether the role of myocardial parasympathetic nerve contributes to ventricular arrhythmias after MI. The autonomic nervous system plays a critical role in the genesis and maintenance of ventricular tachyarrhythmias.^49^ Sympathetic activation and parasympathetic dysfunction are known to accompany MI and increase the risk of sudden cardiac death.^49^ It is widely recognized that parasympathetic innervation is sparse in the myocardium compared with sympathetic nerves.^50^ Indeed, previous studies have shown that border zone tissue was essentially devoid of parasympathetic nerves and was hyperinnervated by sympathetic nerves between 1 and 28 days in rats after MI.^51^ Thus, although E2 can act centrally to enhance parasympathetic efferent tone in non‐infarcted rats,^52^ the antiarrhythmic effects of E2 by increasing myocardial parasympathetic innervation were expected unlikely. Taken together, regardless of the relative importance of each of these factors, all of the E2‐caused sympathetic and parasympathetic changes are compatible with our understanding of their protective effects against ventricular arrhythmias.

### 4.4 Conclusions

These data show that the E2 plays an important role in the sympathetic reinnervation through a superoxide‐dependent macrophage polarization after infarction. E2 treatment may be a new strategy to prevent ventricular arrhythmias after an infarction. Further studies on the specific role that E2 plays in the myocardium may contribute increase our understanding of its neuroprotective actions and potentially lead to the development of novel neuroprotective therapies.

## CONFLICTS OF INTEREST

The authors confirm that there are no conflicts of interest.

## AUTHOR CONTRIBUTION

**Cheng‐Che Lee:** Formal analysis (supporting); Methodology (supporting); Project administration (supporting); Writing – original draft (supporting); Writing – review & editing (supporting). **Syue‐yi Chen:** Conceptualization (supporting); Data curation (supporting); Formal analysis (supporting); Project administration (supporting); Writing – review & editing (supporting). **Tsung‐Ming Lee:** Conceptualization (lead); Data curation (supporting); Formal analysis (supporting); Funding acquisition (lead); Investigation (lead); Methodology (supporting); Project administration (supporting); Resources (lead); Supervision (lead); Validation (lead); Writing – original draft (supporting); Writing – review & editing (supporting).

## Supporting information

Supplementary Material

## Data Availability

The data that support the findings of this study are available from the corresponding author upon reasonable request.
