# Supplementary material for: 17β‐Oestradiol facilitates M2 macrophage skewing and ameliorates arrhythmias in ovariectomized female infarcted rats
Source: J Cell Mol Med. 2022 May 5;26(12):3396–409. doi: 10.1111/jcmm.17344 (PMC9189348; doi:10.1111/jcmm.17344)
Supplement: Supplementary file 1 — Supplementary Material [file JCMM-26-3396-s001.docx]

#### Supplementary Methods

***Hemodynamics and infarct size measurements***

After the rats had been anesthetized with Zoletil-xylazine (20 mg/kg-9 mg/kg) intraperitoneally at the end of the study, the hemodynamic parameters were measured. A polyethylene Millar catheter was inserted into the left ventricle and connected to a transducer (Model SPR-407, Miller Instruments, Houston, TX). LV systolic and diastolic pressures were recorded as the mean of five consecutive pressure cycle measurements as described previously (1). In addition, the maximum rates of rise (+dP/d*t*) and decrease (-dP/d*t*) in LV pressure were also calculated. Electrophysiological tests were performed after the arterial pressure measurements had been made. After the electrophysiological tests had been completed, the atria and right ventricles of the hearts were trimmed off, and the left ventricles were rinsed in cold physiological saline, weighed, and flash frozen in liquid nitrogen. A section taken from the equator of the LV, fixed in 10% formalin, embedded in paraffin, and stained with hematoxylin and eosin and trichrome to determine the size of the infarct size as previously described (2).

***Western Blot Analysis of iNOS, IL-10, and NGF***

iNOS and IL-10 were evaluated in samples obtained from the border zone on day 3, and NGF was evaluated in samples obtained from the remote zone (>2 mm away from the infarct) on day 28. The myocardium was homogenized in three volumes of Tris-buffered saline containing phosphatase and protease inhibitors (25 mM Tris-HCl, pH 7.4, 1 mM EDTA, 1 mM phenylmethylsulfonyl fluoride, 30 mM β-glycerophosphate, 1 mM EGTA, 150 mM NaCl, 5 mM sodium pyrophosphate, 30 mM sodium fluoride). The homogenates were centrifuged for 10 minutes at 500×g and 4°C and 2000×g for 10 minutes, respectively. After separation of 20 μg of protein using 10% SDS-PAGE, the proteins were electrotransferred onto a nitrocellulose membrane and incubated with antibodies. After rinsing the membrane with a blocking solution, it was incubated for 2 hours at room temperature. Antigen-antibody complexes were detected using 5-bromo-4-chloro-3-indolyl-phosphate and nitroblue tetrazolium chloride (Sigma). A scanning densitometer was then used to volume-integrated films within the linear range of the exposure. All experiments were repeated three times, and the results were expressed as mean values.

The following primary antibodies were used in this study: iNOS (Cell Signaling Technology, Danvers, MA, USA), IL-10 (R& D systems, Abingdon, UK), NGF (Chemicon), and β-actin (Sigma-Aldrich). Experiments were replicated three times and results expressed as the mean value.

***Real-time RT-PCR of IL-6, IL-1β, iNOS, CD206, IL-10, and NGF***

Real-time quantitative RT-PCR was performed from samples obtained from the border zone with the TaqMan system (Prism 7700 Sequence Detection System, PE Biosystems) at day 3 as previously described (3). We analyzed the expression of gene markers for M1 (*IL-6, IL-1β, iNOS*) and M2 (*CD206, IL-10*) macrophages. Besides, to further confirm the gene changes of *NGF,* the gene expression was assessed from the remote zone 28 days after infarction. Standard curves were plotted with the threshold cycles versus log template quantities. After initial denaturation, amplification was performed at 95°C (10 s) 60°C (5 s) 72°C (10 s) for 45 cycles. Fold change was normalized against *cyclophilin*, a housekeeping gene.

Forward and reverse sequences of RT-PCR primers

|  | Primer sequence (5’-3’) |  |  |
| --- | --- | --- | --- |
| *Target Gene* | Forward | Reverse |  |
| *IL-6* | CCAGTTGCCTTCTTGGGACTGATG | ATTTTCTGACCACAGTGAGGAATG | |
| *IL-1β* | ATGGCAACTGTCCCTGAACTCAACT | CAGGACAGGTATAGATTCAACCCCTT | |
| *iNOS* | TCACCTTCGAGGGCAGCCGA | TCCGTGGCAAAGCGAGCCAG | |
| *CD206* | TGGGTTTGCTGAAGAAGAGAA | CATGTGATAAGTGACAAATGCTTG | |
| *IL-10* | GGTTGCCAAGCCTTGTCAGAA | GCTCCACTGCCTTGCTTTTATT | |
| *NGF* | CACACTGAGGTGCATAGCGT | TGATGACCGCTTGCTCCTGT | |
| *Cyclophilin* | ATGGTCAACCCCACCGTGTTCTTCG | CGTGTGAAGTCACCACCCTGACACA | |

**Supplementary Table 1. Morphometry and hemodynamics in all infarcted rats at the end of study**

|  | **Sham** |  | **Infarction treated with** | | | |
| --- | --- | --- | --- | --- | --- | --- |
| **Parameters** | **intact** |  | **intact** | **OVX** | **OVX/E2** | **OVX/E2/SIN** |
| No. of rats | 10 |  | 18 | 21 | 22 | 24 |
| Body weight, g | 231 ± 20 |  | 233 ± 12 | 294 ± 21*† | 238 ± 15 | 237 ± 15 |
| HR, bpm | 402 ± 20 |  | 403 ± 18 | 418 ± 19*† | 408 ± 18 | 402 ± 20 |
| LVESP, mm Hg | 105 ± 6 |  | 102 ± 7 | 105 ± 10 | 102 ± 10 | 98 ± 11 |
| LVEDP, mm Hg | 5 ± 4 |  | 16 ± 5*** | 17 ± 4*** | 18 ± 4*** | 19 ± 5*** |
| LVW/BW, mg/g | 3.28 ± 0.27 |  | 3.41 ± 0.55 | 3.72 ± 0.82 | 3.63 ± 0.45 | 3.82 ± 0.72 |
| RVW/BW, mg/g | 0.42 ± 0.16 |  | 0.60 ± 0.20* | 0.67 ± 0.21* | 0.69 ± 0.23* | 0.73 ± 0.21* |
| +dp/d*t*, mm Hg/sec | 7425 ± 227 |  | 2952 ± 265** | 4022 ± 298**† | 3144 ± 279** | 2982 ± 284** |
| -dp/d*t*, mm Hg/sec | 4762 ± 282 |  | 2678 ± 282* | 3165 ± 279*† | 2762 ± 249* | 2578 ± 277* |
| Infarct size, % | … |  | 31.5 ± 7.2 | 33.2 ± 8.5 | 30.7 ± 7.9 | 32.2 ± 8.9 |

Values are mean ± SD. BW, body weight; E2, estradiol; HR, heart rate; LVEDP, left ventricular end-diastolic pressure; LVESP, left ventricular end-systolic pressure; LVW, left ventricular weight; OVX, ovariectomy; RVW, right ventricular weight.

**P* < 0.05, ***P* < 0.01, ****P* < 0.001 compared with sham;

†*P* < 0.05 compared with infarcted groups treated with intact, OVX/E2, and OVX/E2/SIN.

**References.**

1. Lee TM, Harn HJ, Chiou TW, Chuang MH, Chen CH, Lin PC, Lin SZ. [Targeting the pathway of GSK-3β/nerve growth factor to attenuate post-infarction arrhythmias by preconditioned adipose-derived stem cells.](https://www.ncbi.nlm.nih.gov/pubmed/28130118) J Mol Cell Cardiol. 2017 Mar;104:17-30.

2. [Zhang JJ](https://www.ncbi.nlm.nih.gov/pubmed/?term=Zhang%20JJ%5BAuthor%5D&cauthor=true&cauthor_uid=21030494), [Xu ZM](https://www.ncbi.nlm.nih.gov/pubmed/?term=Xu%20ZM%5BAuthor%5D&cauthor=true&cauthor_uid=21030494), [Zhang CM](https://www.ncbi.nlm.nih.gov/pubmed/?term=Zhang%20CM%5BAuthor%5D&cauthor=true&cauthor_uid=21030494), [Dai HY](https://www.ncbi.nlm.nih.gov/pubmed/?term=Dai%20HY%5BAuthor%5D&cauthor=true&cauthor_uid=21030494), [Ji XQ](https://www.ncbi.nlm.nih.gov/pubmed/?term=Ji%20XQ%5BAuthor%5D&cauthor=true&cauthor_uid=21030494), [Wang XF](https://www.ncbi.nlm.nih.gov/pubmed/?term=Wang%20XF%5BAuthor%5D&cauthor=true&cauthor_uid=21030494), [Li C](https://www.ncbi.nlm.nih.gov/pubmed/?term=Li%20C%5BAuthor%5D&cauthor=true&cauthor_uid=21030494). Pyrrolidine dithiocarbamate inhibits nuclear factor-κB pathway activation, and regulates adhesion, migration, invasion and apoptosis of endometriotic stromal cells. [Mol Hum Reprod.](https://www.ncbi.nlm.nih.gov/pubmed/?term=Pyrrolidine+dithiocarbamate+inhibits+nuclear+pathway+activation+%2C+2011) 2011;17:175-81.

3. Lee TM, Chen CC, Chang NC. [Cardiac sympathetic hyperinnervation in deoxycorticosterone acetate-salt hypertensive rats.](https://www.ncbi.nlm.nih.gov/pubmed/22507072) Clin Sci (Lond). 2012 Oct;123(7):445-57.

4. [Bélichard, P](http://www.ncbi.nlm.nih.gov/pubmed?term=%22B%C3%A9lichard%20P%22%5BAuthor%5D).; [Savard, P](http://www.ncbi.nlm.nih.gov/pubmed?term=%22Savard%20P%22%5BAuthor%5D).; [Cardinal, R](http://www.ncbi.nlm.nih.gov/pubmed?term=%22Cardinal%20R%22%5BAuthor%5D).; [Nadeau, R](http://www.ncbi.nlm.nih.gov/pubmed?term=%22Nadeau%20R%22%5BAuthor%5D).; [Gosselin, H](http://www.ncbi.nlm.nih.gov/pubmed?term=Gosselin%20H%5BAuthor%5D&cauthor=true&cauthor_uid=8294707).; [Paradis, P](http://www.ncbi.nlm.nih.gov/pubmed?term=Paradis%20P%5BAuthor%5D&cauthor=true&cauthor_uid=8294707).; [Rouleau, J.L](http://www.ncbi.nlm.nih.gov/pubmed?term=Rouleau%20JL%5BAuthor%5D&cauthor=true&cauthor_uid=8294707). Markedly different effects on ventricular remodeling result in a decrease in inducibility of ventricular arrhythmias. J. Am. Coll. Cardiol. 23:505-513; 1994.
